# Analysis of the occurrence of hemophagocytic lymphohistiocytosis (HLH) features in patients with sepsis: a prospective study

**DOI:** 10.1038/s41598-021-90046-4

**Published:** 2021-05-18

**Authors:** Dominik Bursa, Agnieszka Bednarska, Andrzej Pihowicz, Marcin Paciorek, Andrzej Horban

**Affiliations:** 1 Department of Adults’ Infectious Diseases, Medical University of Warsaw, 61 Żwirki i Wigury St., 02-091 Warsaw, Poland; 2 Hospital for Infectious Diseases, 37 Wolska St., 01-201 Warsaw, Poland

**Keywords:** Bacterial infection, Autoimmune diseases, Inflammatory diseases

## Abstract

HLH syndrome may mimic sepsis but requires entirely different treatment. The aim of the study was to assess the occurrence of HLH features in patients with sepsis and the influence these exert on the patients’ prognosis. The prospective study included 108 patients with suspected sepsis who were routinely evaluated according to HLH criteria. They were divided into group I (SOFA = 2, n = 57) and group II (SOFA ≥ 3, n = 51). Four patients were excluded from analysis: 1 with real HLH, 2 with Still’s disease and 1 with lymphoma. The median (IQR) concentration of ferritin was 613.4 (850.3) ng/mL, however 6 patients revealed a remarkedly high ferritin concentration > 3000 ng/mL, including 2 with ferritin > 10,000 ng/mL. In total, 21 patients met ≥ 4/8 HLH criteria and were found to have sepsis with HLH-like syndrome (SHLS). Out of these, 19 responded to antimicrobials, 2 died due to infection. The sepsis patients presented with the following HLH criteria: fever (95.2%), hyperferritinemia (57.3%), splenomegaly (43.4%), reduced NK cell activity (35.2%), high sCD25 activity (27.4%) and rarely: hypertriglyceridemia (14.4%), duopenia (5.8%), hypofibrinogenemia (1.9%). Although group II patients had higher odds for SHLS presentation (OR 3.26, *p* = 0.026) and for death (OR 14.3, *p* = 0.013), SHLS occurrence had no impact on the risk of death (OR 0.77, *p* = 0.75). Sepsis patients can present with SHLS exclusively due to severe infection. Duopenia, hypertriglyceridemia, hypofibrinogenemia and high level of sCD25 are unusual in sepsis and might indicate real HLH syndrome. Hyperferritinemia, even as high as in real HLH syndrome, can occur in sepsis patients.

## Introduction

In the course of sepsis, the immune system is activated and its overexpression leads to organ dysfunction. In 2016, a new definition of sepsis based on the SOFA (*Sepsis-related Organ Failure Assessment*) score was introduced^1^. Since that time, sepsis has been defined as a life-threatening organ dysfunction caused by a dysregulated host response to infection. Hemophagocytic lymphohistiocytosis (HLH) is a life-threatening condition of a generalized inflammatory response that results from the pathological activation of the immune system^2^. HLH syndrome can be almost identical to sepsis; therefore, it may contribute to the deaths of patients with an incorrect diagnosis of sepsis^3,4^. According to its reported incidence, the rate varies from 1 to 10 in 1,000,000 people, but this figure is probably underestimated^5–8^. HLH diagnostic criteria have been developed in children and extrapolated to adults, hence their accuracy in the latter seems to be somewhat limited. In adult patients, HLH is mostly secondary, and is caused by three main factors: infections, autoimmune diseases and neoplasms^9^. Secondary HLH, especially infection-related, almost entirely meets the definition of sepsis, as an abnormal inflammatory syndrome due to infection and leading to organ dysfunction resulting from a “cytokine storm”. Distinguishing the two syndromes is challenging but extremely important, because patients with HLH syndrome require aggressive immunosuppression.

The aim of the study was to assess the occurrence of HLH features in patients with sepsis and the influence these exert on the patients’ prognosis as well as on the assessment of parameters—allowing to differentiate sepsis with HLH features from real HLH syndrome. Moreover, the study concerned the evaluation of ferritin as a marker distinguishing sepsis and real HLH syndrome.

## Methods

Our prospective, non-interventional study enrolled patients with suspected sepsis or septic shock over 18 years of age. The patients were hospitalized in the Hospital for Infectious Diseases in Warsaw, in the Department of Adults’ Infectious Diseases specializing in treating severe infections, sepsis and infections of the nervous system, as well as in the Intensive Care Unit intended for unstable, critically ill patients with a similar profile to the one mentioned.

Patients were admitted to our centre from other hospitals in the Mazowieckie voivodship (province), mainly form Internal, Neurological, Neurosurgical Wards and from Emergency Departments.

**Figure 1:**
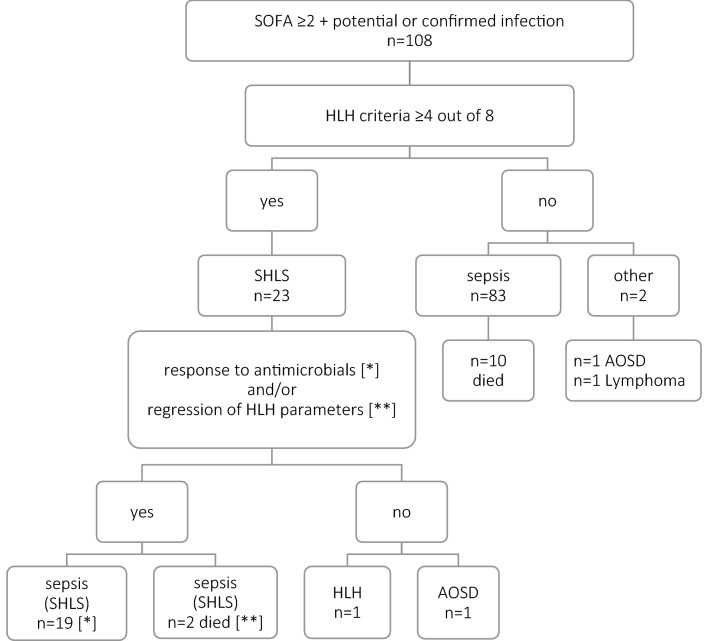
The results of routine assessment of HLH syndrome criteria in the patients with suspected sepsis (n = 108). SHLS – sepsis with HLH like syndrome, HLH—Hemophagocytic lymphohistiocytosis, AOSD – adult-onset Still’s disease.

The study was conducted from October 2016 to July 2018. Sepsis was recognized on the basis of organ dysfunction arising from dysregulated host response to infection^1^. The organ dysfunction was assessed according to the SOFA score amounting to ≥ 2. Infection was defined as potential or microbiologically confirmed. The patients were divided into two groups according to the SOFA score to assess the influence of sepsis severity on HLH feature presentation and the death of the patients: group I with SOFA = 2 and group II with SOFA ≥ 3. The criteria for HLH syndrome were evaluated in all sepsis patients in accordance with the HLH-2004 Diagnostic Protocol established by the Histiocyte Society^10^. HLH diagnosis is based on the fulfilment of at least 5 out of the 8 criteria listed in Table 1. Given the fact that in the early stage of HLH, patients can present with only 4 out of the 8 criteria, this threshold was found to be appropriate for including patients with HLH features^2^. Patients with sepsis who presented with HLH features (≥ 4/8) were considered to have sepsis with HLH-like syndrome (SHLS). The patients with SHLS who responded to antimicrobials and showed regression of met HLH criteria had hyperinflammatory reaction and a cytokine storm exclusively due to infection, while those who neither responded to the treatment, nor showed regression of the met HLH criteria—had real HLH syndrome—Fig. 1. The latter patients were enrolled for bone marrow aspiration. The presentation of HLH features due to sepsis (SHLS) or the death of the patients were clinical endpoints.

**Table 1:** HLH syndrome criteria according to the HLH-2004 diagnostic and therapeutic guidelines. Adapted from^10^.

HLH diagnosis is based on the presence of at least 5 out of the 8 following criteria:
Fever > 38.5
Splenomegaly
Cytopenia ≥ 2 cell lines:
Haemoglobin < 9.0 g/L
Neutrophils < 1.0/mL
Platelets < 100 G/L
Triglycerides > 265 mg/dL (3.0 mmol/L) or fibrinogen < 150 mg/dL
Ferritin > 500 ng/mL
Hemofagocytosis (in biopsy of bone marrow, spleen, lymph node or liver)
Low/absent NK-cell activity
Soluble CD25 (soluble IL2-receptor, sCD25) > 2,400 U/mL

*Material* blood was drawn for basic laboratory tests directly after admission—Table 2. Activity of sCD25 was tested by enzyme-linked immunosorbent assay (ALPCO, Human Soluble Interleukin-2 Receptor, sIL-2R ELISA). To determine NK cell activity, lymphocytes were isolated from the patients and incubated with human erythrocytic leukaemia cells and then the NK cells were introduced into a flow cytofluorimeter (FACSCalibur, Beckton-Dickinson). Normal NK cell level: 6–18%. Normal NK cells activity: 4–15%. Additionally, an abdomen ultrasound was performed on the first working day to assess the spleen and liver size.

**Table 2:** The set of routine blood tests on admission in patients with suspected sepsis.

Complete blood count (CBC)
Aspartate and alanine aminotransferase (AST and ALT)
Gamma-glutamyl transferase (GGT)
Alkaline phosphatase (ALP)
Bilirubin, glucose
Lactate dehydrogenase (LDH)
Creatine phosphokinase (CPK)
C-reactive protein (CRP)
Procalcitonin (PCT)
Lactic acid
Interleukin 6 (IL-6)
Creatinine, blood urea nitrogen (BUN)
Ferritin, serum iron (Fe), total iron binding capacity (TIBC)
D*-*dimer, fibrinogen
Activated partial thromboplastin time (APTT)
Cholesterol, triglycerides
NK-cell activity, sCD25

### Statistical analysis

Group comparison was analysed with a non-parametric test (Mann–Whitney U test) and a parametric test (T-St udent test), depending on the data normality distribution (Shapiro–Wilk test). To assess the statistical significance between the qualitative variables, the Chi^2^ test was used. Logistic regression analysis was performed to determine factors influencing the occurrence of the clinical endpoints (presentation of the HLH features exclusively due to sepsis or the death of the patients). The level of statistical significance was determined with the value of *p* < 0.05. All the analyses were performed using Statistica version 13.3, StatSoft, with a medical add-on.

### Bioethical issues

We confirm that all the experimental protocols were approved by the Bioethical Committee of the Medical University of Warsaw, No. KB/180/2016 of 06.09.2016. We confirm that informed consent was obtained from all participants, as well as that all research was performed in accordance with relevant guidelines and regulations.

## Results

The study group consisted of 108 patients, in group I (SOFA = 2) there were 57 patients (52.8%), while in group II (SOFA ≥ 3) 51 patients (47.2%). Despite initially suspected sepsis, 4 patients (3.7%) from group I were eventually diagnosed with another disease and excluded from the analysis. These were: a 56-year-old male with real HLH syndrome (6 HLH criteria, ferritin 1,927 ng/mL, SOFA = 2) who was referred to the Haematological Department, a 59-year-old female and a 22-year-old male with adult-onset Still’s disease—AOSD (4 HLH criteria, ferritin 97,221 ng/mL, SOFA = 2 and 3 HLH criteria, ferritin 3983 ng/mL, SOFA = 2) who were eventually referred to the Rheumatological Department. The fourth patient presented with an advanced neoplasm (lymphoma) and was disqualified from chemotherapy. He died due to infection (male, 58 years old, 4 HLH criteria, ferritin 11,385 ng/mL, SOFA = 2).

In the studied group, sepsis was a result of the following infections (one or more): pneumonia (n = 29), bacterial meningitis and brain abscesses (n = 27), skin and subcutaneous tissue infections (n = 17), gastrointestinal infections (n = 16), urinary tract infections (n = 10), spondylodiscitis (n = 4), infective endocarditis (n = 3), malaria/dengue fever (n = 3), HIV-related infections (n = 3).

### Occurrence of HLH features in sepsis

In the sepsis patients, the median Md (interquartile range, IQR) ferritin concentration was 613.4 (850.3) ng/mL, however, 6 sepsis patients presented with a remarkably high ferritin concentration, including 4 with results in the range of 3000–10,000 ng/mL, and 2 with hyperferritinemia 17,725 and 17,284 ng/mL. The most common HLH criteria presented by the patients with sepsis were: fever (95.2%), hyperferritinemia (57.3%) and splenomegaly (43.4%). Every third patient had reduced NK lymphocyte activity (35.2%) and high sCD25 activity (27.4%). Hypertriglyceridemia, duopenia and hypofibrinogenemia were rarely observed, with a frequency of 14.4%, 5.8% and 1.9%, respectively—Table 3.

**Table 3:** General characteristics and HLH features presentation in the studied group (n = 104, Chi2 test, Mann–Whitney U test ).

Parameters	Group I SOFA = 2 n/Md(IQR)	Group II SOFA ≥ 3 n/Md(IQR)	*P*-value	In total group n(%)/Md(IQR)
General group characteristics				
Patients with suspected sepsis	57	51	–	108
Patients with sepsis	**53**	**51**	–	104
Patients with septic shock	–	9	–	9
Patients with non-infectious syndromes [including HLH]	4 [1]	–	–	4 [1]
**Patients with SHLS**				
≥ 4/8HLH)	6	15	0.02	21
(≥ 5/8HLH)	1	3	NS	4
Ferritin (ng/mL)	413.7 (539.1)	820.8 (1042.4)	0.002	613.4 (850.3)
Age (years)	58 (34)	65 (37)	NS	61 (37.5)
Gender: male/female	22/31	33/18	0.02	55/49
Patients who died	1	11	0.002	12
Community acquired infection	49	45	NS	94
Nosocomial infection	4	6	NS	10
Diabetes mellitus	11	12	NS	23
Splenectomy	0	2	NS	2
Alcohol addiction	1	5	NS	6
Steroid therapy	10	20	0.02	30
Hydrocortisonum	2	4	NS	6
Dexamethasone	8	16	0.048	24
**Etiological factors**	40/53	36/51	NS	76/104 (73.1%)
Streptococcus pneumoniae	6	14	–	20
Streptococcus pyogenes	11	1	–	12
Streptococcus agalactiae	1	0	–	1
Enterococcus spp	1	0	–	1
Streptococcus mitis	1	0	–	1
MSSA	4	4	–	8
MRSA	1	2	–	3
Coagulase-negative staphylococci (CNS)	4	0	–	4
Listeria monocytogenes	1	1	–	2
Clostridium difficile	0	2	–	2
Salmonella enteritidis	4	0	–	4
Klebsiella pneumoniae	1	1	–	2
Neisseria meningitidis	1	2	–	3
*Escherichia coli*	3	2	–	5
Stenotrophomonas maltophilia	0	1	–	1
Hemophilus influenzae	1	0	–	1
Plasmodium falciparum	0	3	–	3
Pneumocystis jiroveci (PJP), CMV, HIV	0	1	–	1
Influenza virus	0	1	–	1
Dengue fever virus	0	1	–	1
**HLH criteria in the sepsis**				
Fever [> 38.5]	50/53	49/51	NS	99/104 (95.2%)
Hyperferritinemia [> 500 ng/mL]	23/53	36/50	0.003	59/103 (57.3%)
[> 1000 ng/mL]	8/53	20/50	0.004	28/103 (27.2%)
[> 1500 ng/mL]	6/53	12/50	NS	18/103 (17.5%)
[> 2000 ng/mL]	5/53	6/49	NS	11/102 (10.8%)
[> 5000 ng/mL]	1/53	3/49	NS	4/102 (3.9%)
Splenomegaly	22/53	21/46	NS	43/99 (43.4%)
Hepatomegaly	12/53	14/46	NS	26/99 (26.3%)
Low/absent NK-cell activity	8/23	11/31	NS	19/54 (35.2%)
High sCD25 activity [> 2400 U/mL]	8/39	12/34	NS	20/73 (27.4%)
Hypertriglyceridemia [> 3.0 mmol/L]	5/53	10/51	NS	15/104 (14.4%)
Duopenia [Haemoglobin < 9.0 g/L and/or Neutrophils < 1.0/mL and/or Platelets < 100 G/L]	0/53	6/51	0.01	6/104 (5.8%)
Hypofibrinogenemia [< 150 mg/dL]	0/53	2/51	NS	2/104 (1.9%)
Hypofibrinogenemia + hypertriglyceridemia	5/53	11/51	NS	16/104 (15.4%)

N, number of patients; NS, not statistically significant; SHLS, sepsis with HLH like syndrome; HLH, Hemophagocytic lymphohistiocytosis; MSSA, methicillin-sensitive *Staphylococcus aureus*; MRSA, Methicillin-resistant *Staphylococcus aureus*; sCD25, Soluble CD25; CMV, Cytomegalovirus; HIV, Human immunodeficiency virus.

As many as every fifth patient with sepsis (n = 21, 20.2%) presented with SHLS. Most of these patients (n = 19/21) responded to antimicrobials (n = 6/19 received also steroids), nevertheless, the remaining 2 patients died—Fig. 1. Those who died showed a typical course of severe infection with evident regression of HLH criteria and/or their parameters and initially met only a borderline number of HLH criteria, namely 4. They did not receive steroids.

The severity of sepsis, i.e. belonging to group I or II, had an impact on the presentation of the HLH features (SHLS). The patients in group II showed SHLS 2.5-times more often (n = 15 vs. n = 6, *p* = 0.02) and had 3.3-fold higher odds (*p* = 0.026) of revealing SHLS than group I patients—Tables 3, 4. Furthermore, it was found that among the qualitative variables, all HLH criteria but hypofibrinogenemia were strongly related to SHLS presentation. Out of the quantitative variables, triglycerides and sCD25 were found to be independent predictors of SHLS occurrence (OR 2.93, *p* = 0.002 and OR 1.001, *p* = 0.008, respectively)—Table 4.

**Table 4:** Variables associated with the occurrence of SHLS (n = 104. Univariate logistic regression and multivariate stepwise regression—for quantitative variables with *p* < 0.2 for the model; for qualitative variables—model unavailable). OR, odds ratio; 95Cl, 95% Confidence Interval; sCD25, Soluble CD25.

Variables	Univariate regression				Multivariate regression			
Quantitative	OR	*P*	− 95%	95%	OR	*P*	− 95%	95%
Age	0.994	0.6139	0.972	1.017	–			
Glasgow coma scale	1.063	0.5312	0.877	1.289	–			
SOFA score	1.126	0.0974	0.979	1.296	–			
Hemoglobin	0.866	0.2447	0.680	1.103	–			
Neutrophils	0.928	0.0778	0.855	1.008	–			
Platelets	*0.993*	*0.0149*	*0.988*	*0.999*	–			
Triglycerides	*3.394*	*0.0000*	*1.900*	*6.063*	*2.932*	*0.002*	*1.465*	*5.866*
Fibrinogen	0.917	0.4103	0.745	1.128	–			
Ferritin	1.000	0.1266	1.000	1.000	–			
NK cells activity	1.059	0.3657	0.936	1.198	–			
Soluble CD25	*1.001*	*0.0019*	*1.000*	*1.001*	*1.001*	*0.008*	*1.000*	*1.001*
C-reactive protein	1.000	0.8341	0.996	1.003	–			
Procalcitonin	1.000	0.8945	0.995	1.004	–			
Lactic acid	1.007	0.9692	0.713	1.421	–			
Interleukin 6	1.000	0.4229	1.000	1.000	–			
Bilirubin	*1.052*	*0.0147*	*1.010*	*1.095*	–			
Creatinine	1.003	0.2902	0.997	1.010	–			
Serum iron (Fe)	1.050	0.2052	0.974	1.132	–			
Total iron binding capacity (TIBC)	0.980	0.4318	0.933	1.030	–			
D-dimer	1.000	0.2006	1.000	1.000	–			
**Qualitative**								
Gender (male vs. female)	*3.610*	*0.0213*	*1.210*	*10.768*	–			
Steroids use	0.983	0.9752	0.341	2.836	–			
Dexamethasone	0.741	0.6246	0.223	2.460	–			
Hydrocortisone	2.079	0.4176	0.354	12.202	–			
Sepsis (group II vs. I)	*3.264*	*0.0260*	*1.152*	*9.249*	–			
Diabetes mellitus	1.128	0.8342	0.364	3.500	–			
Alcohol addiction	2.079	0.4176	0.354	12.202	–			
Splenectomy	4.100	0.3258	0.246	68.417	–			
Splenomegaly	*12.720*	*0.0001*	*3.427*	*47.215*	–			
Hepatomegaly	*3.523*	*0.0153*	*1.273*	*9.746*	–			
Duopenia [≥ 2/3 lines]	*9.529*	*0.0129*	*1.613*	*56.289*	–			
Hypertriglyceridemia [> 3.0 mmol/L]	*21.725*	*0.0000*	*5.803*	*81.327*	–			
Hypofibrinogenemia [< 150 mg/dL]	4.100	0.3258	0.246	68.417	–			
Hypofibrinogenemia + hypertriglyceridemia	*17.160*	*0.0000*	*4.939*	*59.618*	–			
Hyperferritinemia [> 500 ng/mL]	*22.051*	*0.0032*	*2.826*	*172.088*	–			
[> 1000 ng/mL]	*7.258*	*0.0002*	*2.556*	*20.610*	–			
[> 1500 ng/mL]	*4.431*	*0.0081*	*1.473*	*13.330*	–			
[> 2000 ng/mL]	*6.600*	*0.0050*	*1.769*	*24.618*	–			
[> 5000 ng/mL]	4.444	0.1489	0.586	33.692	–			
Low/absent NK-cell activity	0.633	0.5401	0.146	2.736	–			
High sCD25 activity [> 2400 U/mL]	*14.400*	*0.0000*	*3.988*	*52.000*	–			

The level of statistical significance was determined with the value of (*p* < 0.05).

### Factors influencing death

A difference was also found between group I and group II patients with respect to the number of deaths. In group I, only 1 patient died, while in group II as many as 11 (*p* = 0.002) passed away—Table 3. The odds of death in group II were 14 times higher than in group I, *p* = 0.01. Moreover, belonging to group II was found to be an independent predictor of death (OR 11.2, *p* = 0.02)—Table 5. In the entire study group, variables related to the risk of death were: SOFA score as well as lactic acid, fibrinogen, TIBC and lactate dehydrogenase (LDH). Lactic acid was the only parameter from the current definition of sepsis which increased the probability of death in a statistically significant way. In multivariate regression, the SOFA score and LDH had a significant impact on the occurrence of death. Mortality was not affected by the SHLS presentation—Table 5.

**Table 5:** Variables associated with the death of the patients (n = 104. Univariate logistic regression and multivariate stepwise regression – for quantitative variables with *p* < 0.05 for the model; for qualitative with *p* < 0.2 for the model).

Variables	Univariate regression				Multivariate regression			
Quantitative	OR	*P*	− 95%	95%	OR	*P*	− 95%	95%
Age	1.022	0.1579	0.991	1.054	–			
SOFA score	*1.465*	*0.0006*	*1.178*	*1.822*	*1.464*	*0.035*	*1.027*	*2.089*
Hemoglobin	0.770	0.0885	0.570	1.040	–			
Neutrophils	0.930	0.1754	0.836	1.033	–			
Platelets	0.996	0.2291	0.990	1.002	–			
Triglycerides	0.950	0.8683	0.521	1.733	–			
Fibrinogen	0.715	0.0208	0.538	0.950	–			
Ferritin	1.000	0.1577	1.000	1.000	–			
NK cells activity	0.882	0.2512	0.713	1.093	–			
Soluble CD25	1.000	0.4620	1.000	1.001	–			
C-reactive protein	1.000	0.9317	0.996	1.004	–			
Procalcitonin	0.999	0.8355	0.992	1.007	–			
Lactic acid	*1.465*	*0.0331*	*1.031*	*2.082*	–			
Interleukin 6	1.000	0.0684	1.000	1.000	–			
Bilirubin	0.977	0.5212	0.910	1.049	–			
Lactate dehydrogenase (LDH)	*1.003*	*0.0006*	*1.001*	*1.005*	*1.003*	*0.048*	*1.000*	*1.006*
Creatinine	1.004	0.2817	0.997	1.011	–			
Serum iron (Fe)	1.076	0.1143	0.982	1.179	–			
Total iron binding capacity (TIBC)	*0.836*	*0.0056*	*0.736*	*0.949*	–			
D-dimer	1.000	0.7939	1.000	1.000	–			
**Qualitative**								
Gender (male vs. female)	1.667	0.4114	0.493	5.638	–			
Steroids use	1.269	0.7157	0.352	4.580	–			
Dexamethasone	0.636	0.5779	0.130	3.127	–			
Hydrocortisone	4.400	0.1104	0.714	27.131	–			
HLH ≥ 4 (SHLS)	0.768	0.7469	0.155	3.806	–			
HLH (-NK,-sCD25, -BMA) ≥ 4	0.838	0.8729	0.097	7.267	–			
Sepsis (group II vs. I)	*14.300*	*0.0125*	*1.772*	*115.41*	*11.243*	*0.025*	*1.348*	*93.782*
Diabetes mellitus	0.676	0.6305	0.137	3.330	–			
Alcohol addiction	4.400	0.1104	0.714	27.131	–			
Splenomegaly	0.124	0.0525	0.015	1.023	–			
Hepatomegaly	0.284	0.2445	0.034	2.363	–			
Duopenia [≥ 2/3 lines]	*9.889*	*0.0099*	*1.734*	*56.397*	4.625	0.091	0.785	27.246
Hypertriglyceridemia [> 3.0 mmol/L]	1.215	0.8143	0.239	6.188	–			
Hypofibrinogenemia [< 150 mg/dL]	8.273	0.1450	0.483	141.82	–			
Hypofibrinogenemia + hypertriglyceridemia	2.026	0.3341	0.484	8.484	–			
Hyperferritinemia [> 500 ng/mL]	2.460	0.1979	0.625	9.685	–			
[> 1000 ng/mL]	2.112	0.2378	0.610	7.307	–			
[> 1500 ng/mL]	1.689	0.4692	0.409	6.981	–			
[> 2000 ng/mL]	1.800	0.4895	0.340	9.534	–			
[> 5000 ng/mL]	2.636	0.4185	0.252	27.603	–			
Low/absent NK-cell activity	2.844	0.2053	0.564	14.341	–			
High sCD25 activity [> 2400 U/mL]	0.346	0.3359	0.040	3.006	–			

The level of statistical significance was determined with the value of (*p* < 0.05).

OR, odds ratio; 95Cl, 95% Confidence interval; sCD25, Soluble CD25; HLH (-NK,-sCD25, -BMA) ≥ 4, met 4 out of the 5 HLH criteria in the sepsis patients (without NK cells activity, sCD25 concentration and bone marrow aspiration).

In total, 30 patients were given steroids in addition to antibiotics. Those patients had bacterial meningitis (dexamethasone 32–40 mg/d, n = 24), septic shock or allergic reaction (hydrocortisone 100–200 mg/d, n = 6). The use of steroids had no influence either on the occurrence of death (*p* = 0.72), or on the presentation of HLH features (≥ 4 and ≥ 5; *p* = 0.98 and *p* = 0.85). Moreover, no relationship between steroid use and patient death was found in sepsis and SHLS subgroups (*p* = 0.41 and *p* = 0.37).

## Discussion

In the study conducted, routine assessment of HLH criteria in all the patients with sepsis made it possible to identify 1 patient with real HLH syndrome (n = 1/108). The aim of the study was not to assess the occurrence of real HLH syndrome—the study group was too small and included patients with sepsis—so a single case of HLH does not affect epidemiology. However, results significantly different from the global epidemiological data were also obtained in the 3rd degree reference centre of Mayo Clinic, where routine diagnostics of HLH syndrome was introduced in all the patients with a systemic inflammatory syndrome, finding that its incidence could reach the level of 1 in 2000 of the patients admitted^11^. The results, therefore, showed that HLH syndrome is not as rare as it seemed, so it is rational to make HLH assessment on a routine basis in all patients with inflammatory syndromes, particularly with sepsis.

Evaluation of HLH parameters was based on the classic diagnostic criteria. Novel parameters characteristic for HLH syndrome, such as cytokine IL-18 and interferon gamma were not included. We attempted to find diagnostic hints in widely available, basic HLH criteria, allowing to distinguish SHLS and real HLH. One of the main parameters in the differential diagnosis of sepsis and HLH syndrome is ferritin. The sepsis patients commonly presented with ferritin concentration exceeding the cut-off point for HLH syndrome, namely 500 ng/ml. In our study median, ferritin was 613.4 (850.3) ng/mL and two patients revealed as high a concentration as 17,725 and 17,284 ng/mL, i.e. values typical for patients with HLH syndrome. In an ICU study involving patients with HLH syndrome, it was found that median ferritin was 17,728 (7689–37,981) µg/L^12^. Furthermore, in an another study, a maximum ferritin value of 9083 µg/L was at 92.5% sensitivity and 91.9% specificity for hemophagocytic lymphohistiocytosis in adult critically ill patients^13^. Our results indicate that even extreme hyperferritinemia can occur in sepsis and the value of this parameter in HLH recognition seems to be limited.

Complex assessment of HLH criteria revealed that even every fifth patient with sepsis presented with HLH features (SHLS) in the course of severe infection—Table 3. This may indicate the coexistence of real HLH in sepsis or the independent similarity of the pathomechanisms of both syndromes. Studies describing the relationship of sepsis and HLH syndrome are not numerous and usually concern selective aspects of the similarities of both syndromes. It was found that an occurrence of some HLH features in sepsis patients is associated with a higher percentage of multiorgan failure and death^3,14–16^. Furthermore, hemophagocytosis in bone marrow, a typical feature of HLH syndrome, might occur in patients with sepsis and multiorgan failure^17^. In our group of patients, a strong correlation was found between the severity of organ dysfunction (SOFA score) and the number of HLH criteria met, while the relationship between the number of HLH criteria and patient death was not proven—Tables 4, 5. Our patients improved on antimicrobial treatment, which supports the thesis that SHLS results from severe infection, or on the other hand that sepsis-related HLH is self-limiting. Certainly, patients with SHLS—despite the response to antimicrobials frequently observed in the group we examined—require thorough monitoring and regular check-ups for the occurrence of real HLH syndrome.

Therefore, the key question seems to be whether it is possible to distinguish SHLS and real HLH syndrome in their early stage on the basis of characteristic laboratory deviations.

The results obtained in our group of sepsis patients were compared with the results of patients with real HLH syndrome from other studies^18–22^—Table 6.

**Table 6:** Prevalence of particular HLH syndrome criteria in the studied group of patients with sepsis (n = 104) and in other studies, including patients with real HLH syndrome (Adapted data^18–22^).

Data source	Patients with hemophagocytic syndrome (HLH) Adapted data^18–22^					The patients with sepsis
HLH criterium	Li et al	Otrocka et al	Delphi consensus	Bergsten et al	Hayden et al	Our group
Cytopenia	87%	85%	T	92%	N/A	5.8%
Hyperferritinemia	87%	85%	T	94%	N/A	57.3%
Splenomegaly	80%	60%	T	89%	N/A	43.4%
Hepatomegaly	65%	N/A		N/A	N/A	26.3%
hypertriglyceridemia	87%	71%	NC	65%	N/A	14.4%
hypofibrinogenemia	61%	38%	NC	75%	N/A	1.9%
Hypertriglyceridemia and hypofibrinogenemia	N/A	N/A	N/A	90%	N/A	15.4%
low/absent NK-cell activity	N/A	36%	LU	71%	N/A	35.2%
high activity of soluble CD25	N/A	77%	LU	97%	100%	27.4%

N/A – not applicable, T – typical for HLH, NC – no consensus, LU – of little use in everyday practice.

When comparing the frequency of particular criteria in our sepsis patients and in patients with real HLH syndrome, it can be seen that the most common deviations in both groups are hyperferritinemia and organomegaly. The decreased NK lymphocyte activity seems to be somewhat similar in both syndromes. The high activity of sCD25, characteristic for patients with HLH syndrome (77–100%), was found only in 27% of the patients with sepsis. Our results are in line with those obtained by Hayden et al.^22^. They observed sCD25 > 2400 U/ml only in 37.5% of the patients with non-HLH conditions, including sepsis, while in 100% of the patients with real HLH. Moreover, the authors concluded that 2515 U/ml is the optimal cut-off for sCD25 for HLH patients (100% sensitivity and 72.5% specificity) and that sCD25 > 10,000 U/mL has 93% specificity for ruling in HLH. In our study, no one with SHLS reached the level of sCD25 ≥ 10,000 U/ml, nevertheless 13 patients exceeded 3000 U/ml; 3 patients reached the level of 5000 U/ml and the maximal sCD25 in SHLS was 9307.3 U/ml. The diagnostic utility of sCD25 is particularly visible in malignancy-associated hemophagocytic syndrome, where the superiority of this parameter over ferritin is significantly manifested^22^.

The main differences in the presentation of both conditions relate to the following parameters: bi-/pancytopenia, hypertriglyceridemia and hypofibrinogenemia. They are common in HLH syndrome and occur in 85–92%, 65–87% and 38–75% of the cases, respectively, while they are rare in sepsis, i.e. they are observed only in 5.8%, 14.4% and 1.9% of the patients, respectively. A clear difference also applies to sCD25, which is a typical HLH parameter less common in sepsis patients—Table 6.

Debaugnies et al. noticed that the main parameters influencing the occurrence of real HLH syndrome are: cytopenia of 3-cell lines (OR 14.8), hypofibrinogenemia (OR 13.5), hypertriglyceridemia > 4 mmol/L (OR 9.6), ferritinemia > 6000 ng/mL (OR 5.8), organomegaly (OR 5.8)^23^. Pham et al. concluded that the following factors have the most significant impact on the occurrence of HLH syndrome: increased ferritin (OR 36), thrombocytopenia (OR 15), duopenia (OR 8)^24^. In our sepsis patients, similar qualitative variables influenced SHLS presentation in univariate logistic analysis, while in multivariate analysis only triglycerides and sCD25 were found to be independent predictors of SHLS occurrence—Table 4.

Given these considerations, uncommon in sepsis disorders, namely duopenia, hypertriglyceridemia and/or hypofibrinogenemia might be early indicators of developing real HLH syndrome in patients with sepsis and SHLS. A similar observation regarding duo/pancytopenia was made by Kapoor et al., who found this deviation a potential feature of HLH in patients with septic shock and multi organ failure syndrome, not responding to the standard treatment protocols^12^.

Castillo et al. put forward the thesis that infection-associated HLH does not represent a separate entity, but falls within the final spectrum of inflammatory response to sepsis^25^. Both conditions share a similar pathophysiology, which creates difficulties in setting a clear border between them. HLH and sepsis are probably a continuum of the same inflammatory reaction^25^. The key question remains at what moment the patient with SHLS can benefit from immunosuppressive therapy. In the light of the results obtained, it seems that the main argument for the initiation of aggressive immunosuppressive treatment in patients with SHLS is the lack of response to antibiotic therapy supported by the occurrence of the characteristic laboratory differences that were revealed.

The study has limitations. The study group was not numerous, the use of steroids could have interfered both HLH occurrence and the influence of SHLS on the patients’ prognosis, however, on the other hand the study was prospective, patients were enrolled and evaluated according to strictly defined criteria and none of the statistically significant correlations was found between steroid use and the above-mentioned variables. Moreover, the study features advantages in the form of clearly selected sepsis patients with HLH features assessed at an acute phase of the infection, which allowed us to find specific differences in laboratory parameters between sepsis and HLH syndrome.

## Conclusions

Every fifth patient with sepsis presents with HLH features, which mainly result from severe infection, as evidenced by a response to antibiotic therapy and regression of HLH criteria.

Duopenia, hypertriglyceridemia, hypofibrinogenemia and high level of sCD25 are uncommon in sepsis and may indicate the HLH syndrome.

Hyperferritinemia occurs in patients with sepsis and is thereby of limited value in differentiating the two syndromes.

HLH and sepsis are a continuum and development of better diagnostic tools is needed in their diagnosis.
